# The Metabolome and Osteoarthritis: Possible Contributions to Symptoms and Pathology

**DOI:** 10.3390/metabo8040092

**Published:** 2018-12-13

**Authors:** Jason S. Rockel, Mohit Kapoor

**Affiliations:** 1 Arthritis Program, University Health Network, Toronto, ON M5T 2S8, Canada; mohit.kapoor@uhnresearch.ca; 2 Division of Genetics and Development, Krembil Research Institute, University Health Network, Toronto, ON M5T 2S8, Canada; 3 Department of Surgery, University of Toronto, Toronto, ON M1C 1A4, Canada; 4 Department of Laboratory Medicine and Pathobiology, University of Toronto, Toronto, ON M1C 1A4, Canada

**Keywords:** metabolomics, precision medicine, osteoarthritis, local, systemic

## Abstract

Osteoarthritis (OA) is a progressive, deteriorative disease of articular joints. Although traditionally viewed as a local pathology, biomarker exploration has shown that systemic changes can be observed. These include changes to cytokines, microRNAs, and more recently, metabolites. The metabolome is the set of metabolites within a biological sample and includes circulating amino acids, lipids, and sugar moieties. Recent studies suggest that metabolites in the synovial fluid and blood could be used as biomarkers for OA incidence, prognosis, and response to therapy. However, based on clinical, demographic, and anthropometric factors, the local synovial joint and circulating metabolomes may be patient specific, with select subsets of metabolites contributing to OA disease. This review explores the contribution of the local and systemic metabolite changes to OA, and their potential impact on OA symptoms and disease pathogenesis.

## 1. Introduction

Osteoarthritis (OA) is a degenerative joint disease characterized by cartilage degradation, synovitis, subchondral bone thickening, and ectopic bone formation in the form of osteophytes [1]. The etiology of OA is unknown. Current approved therapies only provide symptom relief and are not disease-modifying. Ultimately, patients with OA will need joint replacement therapy once pain, function, and quality of life are no longer satisfactory or adequately controlled. A large proportion of patients who undergo joint replacement surgery continue to feel pain and thus have limited improvement to quality of life and function [2]. Thus, defining patients who are good candidates for surgery is important for providing precision care to each individual.

Traditionally, OA has been viewed as a single disease carrying with it a “one size fits all” approach to treatment. However, a number of different patient phenotypes have been determined based on clinical, demographic, and anthropometric characteristics. Risk factors for OA include age, body mass index (BMI), and sex, among others. Individuals over the age of 50, with a body mass index > 30 kg/m^2^, and of the female sex have an increased incidence and risk of OA [3,4,5,6,7]. In addition, individuals with comorbidities such as diabetes have increased incidence and risk of developing OA and may also have accelerated joint pathogenesis as a result [8,9]. Furthermore, some patients present in the clinic with reduced mobility and are pain free, while others can experience severe pain but exhibit limited structural joint disease [10,11,12]. Based on these multiple patient characteristics, it is likely that there are underlying biological differences that can be detected within each patient that would provide insights into tailored care plans.

Considerable effort has been placed into identifying biomarkers of disease incidence and prognosis that could potentially help in identifying new targets for disease modification. The most common biomarkers investigated to date include circulating microRNAs, cytokines, and metabolites. A total of 690 papers can be identified in PubMed up to the end of November 2018 using the search-string “(microRNA OR metabolite OR cytokine) AND osteoarthritis AND biomarker NOT review.” Of them, 646 relate to cytokines, 32 pertain to microRNAs, and 20 focus on metabolites, with some articles having keyword overlap between biomarker types. One of the 20 articles focused on metabolite biomarkers in OA is a non-English review article, two are in vitro studies only, one is a study on healthy individuals, and one discusses cleavage products of cartilage in equine synovial fluid and plasma in response to recombinant equine growth hormone injection. The 15 remaining articles are summarized in Table 1. Overall, this indicates that metabolite research in OA is in a relatively early but growing exploratory state, as the majority of articles (11/15) were published between 2015–2018. As metabolites can be used as biomarkers, circulating metabolites or those in urine may be indicative of underlying pathologies. There are a vast number of metabolite changes that have been identified in both blood and synovial fluid of OA patients [13], suggesting that metabolites could influence the various OA pathological features or associated comorbidities in both the local disease environment and in distant organs.

This review aims to summarize the changes to the metabolome, determined both locally as well as systemically, in relation to OA and associated pathologies. In addition, this review will explore the various OA phenotypes that have been identified and how demographic, anthropometric, and clinical variables can affect not only OA disease incidence and prognosis, but how these variables can modify the metabolome and contribute to symptoms and biological pathologies associated with OA.

## 2. The Local and Systemic Metabolomes of Osteoarthritis

In the local environment, the joint is bathed in synovial fluid, which contains factors that accumulate through release from local tissues and from the systemic circulation through blood vessels found in the synovium. Changes to the nutrient levels in the synovial fluid, including various metabolites, may directly contribute to inflammatory responses in OA leading to joint pathologies [29]. Metabolomic analysis of human synovial fluid has uncovered a variety of metabolites altered in OA compared to healthy controls. These include concentration-dependent changes to select lipid, sugar, and amino acid derivatives, which are associated with OA diagnosis or grade [30,31,32]. In synovial fluid, a variety of metabolites including amino acids, sugars, and metabolites involved in energy production, measured by proton nuclear magnetic resonance (^1^H-NMR), could differentiate horses with septic vs. non-septic joint pathologies [14]. Similarly, in an ovine model of early OA, metabolomic analysis of synovial fluid uncovered six significantly altered metabolites, namely isobutyrate, glucose, uridine, serine, asparagine, and hydroxyproline, which could be used as early OA biomarkers [33]. Two contradictory studies have indicated that human rheumatoid arthritis (RA) and OA are indistinguishable [34] or dipartite [35], based on ^1^H-NMR spectra. However, alternate metabolite detection platforms confirm differences in metabolites from OA and RA synovial fluid [36], suggesting that the OA metabolome could be used for differential diagnosis. Furthermore, patients with low-grade versus high-grade radiographic OA severity (Kellgren Lawrence (KL)1/2 versus KL3/4 [37]) can be discriminated based on a signature of 28 metabolites in synovial fluid as determined by gas chromatography/time of flight mass spectrometry (GC/TOF-MS) [38]. This suggests that the methods of metabolite detection as well as cohorts used are likely to impact biomarker discovery.

Although OA is primarily considered a localized disease, it has become abundantly clear that systemic effects could contribute to OA symptoms and pathology [39]. A subset of eight metabolites that include three branched chain amino acids (BCAAs), three phospholipids, glycine, and creatinine are highly correlated by concentration in the synovial fluid and plasma of OA patients [24], suggesting some degree of potential interaction between the circulating metabolome and the local joint metabolome. Recent metabolomic studies have uncovered a number of metabolites that are dysregulated in the systemic circulation. For instance, in one of the first serum-based metabolomics study of OA in humans, the ratios of individual branched chain amino acids or total BCAAs to histidine concentrations were determined to be significantly different between OA patients and healthy controls [17,25], and subsequently total BCAAs:histidine was found to be predictive of individuals with OA [17]. Further studies show changes to a variety of different lipid species including phosphatidylcholines (PCs), lysophosphatidylcholines (lysoPCs), sphingolipids, and select amino acids including arginine [17,22]. A study of a cohort from Newfoundland, Canada, found that the ratio of total lysoPCs to PCs is predictive of advanced OA leading to total knee replacement in a 10-year follow-up [17]. Sustained changes to the plasma metabolome, particularly selected lysoPC and PC analogues, were also identified in mice fed a high-fat diet compared to a lean or normal chow-fed diet, correlating with acceleration of surgically-induced OA [40]. Furthermore, in overweight and obese individuals, urinary metabolomics was able to distinguish individuals whose OA progressed versus those with stable disease over a span of 18 months [19]. In rat models of OA, changes to amino acid metabolism are also observed in urine [41]. Again in rats, a longitudinal metabolomic analysis of plasma showed that there are significant and time-dependent changes in products of lysine metabolism after surgical induction of OA [42]. This further suggests that amino acid metabolism may contribute to OA pathology or that select amino acid levels could be used as biomarkers of OA incidence and progression.

As there are a large number of metabolic changes that occur systemically in response to OA, it is not surprising that some of these changes also correlate to functional changes in other organs. For instance, select medium- and long-chain acylcarnitine moieties are systemically reduced in patients with OA compared to healthy controls, possibly a result of increased energy demands and/or reduced β-oxidation from impaired carnitine palmitoyltranferase enzyme function, the rate-limiting enzyme for long-chain β-oxidation. These changes also correlate to arterial stiffness, a pathology associated with cardiovascular disease [43] and a comorbidity whose risk is increased in individuals with OA [44,45,46,47,48,49]. OA also increases the risk of developing type 2 diabetes [50], a metabolic disorder on its own. Interestingly, individuals with OA and comorbidities such as diabetes, cardiovascular disease, and obesity, or who have high consumption of fats, have accelerated cartilage degeneration [8,51,52]. In fact, diabetes is an independent risk factor for OA incidence and surgery [53,54]. There is an abundance of information linking diabetes to an increased risk of cardiovascular disease [55]; however, to our knowledge, there is no published literature that has explored whether cardiovascular disease increases the risk of incident OA. Overall, there is a link between and across these diseases that could be explained, in part, by changes in lifestyle associated with each disease. However, underlying biochemical changes in the circulating metabolome are also likely to be altered and could profoundly affect both incidence and progression of each disease. Understanding metabolome links between co-morbidities may help to understand underlying pathologies and provide novel therapeutic avenues to treat multiple diseases concurrently.

## 3. Osteoarthritis Phenotypes and Impact on Metabolome

OA is a heterogeneous disease and incorporates a number of disease phenotypes with similar pathologies. From independent cohort studies, common clinical and anthropometric variables stand out as being key to defining the risk of OA incidence and potential clinical outcomes. These include age, sex, and BMI. Each of these variables can also carry its own metabolic “signature,” which could complicate subsequent analysis. For instance, age, when adjusted for BMI, has a specific serum metabolite signature that differs in males and females [56], confounding how metabolites may be identified in large population-based studies. In addition, a study comparing plasma from individuals with obesity to those with metabolic syndrome identified a signature of metabolites that could differentiate these individuals, which included branched chain amino acids (BCAAs) such as leucine and isoleucine [57]. This suggests that BCAAs from individuals with metabolic syndrome could contribute to OA symptoms or pathologies; however, no such studies, to our knowledge, have investigated this link. Our recent research has determined that stratification of cohorts based on sex and BMI is necessary to uncover the differential plasma metabolite signatures between healthy control and OA patients at TKR, which are heavily biased towards males [58]. This study also identified that select metabolite signatures composed of individual lysoPC and PC analogues are better at predicting OA incidence compared to total aggregates of lysoPCs and PC analogue types. Furthermore, metabolites in plasma alone can classify different OA phenotypes into two major subgroups without any confounders including age, sex, or BMI [59] suggesting that OA disease may have multiple metabolite signatures that could prove useful in precision medicine applications.

Clinical phenotypes have also been identified which include, but are not limited to, pain sensitization or neuropathic pain, muscle strength, BMI, and level of depression [10,11,12]. How each of these phenotypes could influence the metabolome is described below and articles related to each phenotype are summarized in Table 2.

### 3.1. Pain

OA pain is typically classified into neuropathic (nerve damage associated) or nociceptive (inflammatory or tissue damage associated) [102]. A pilot study of pain indicated that neuropathic pain, nociceptive pain, and pain-free individuals can be differentiated using global metabolomic profiling of urine; although, a preliminary signature for each classification was not provided [60]. Furthermore, in a study of women with widespread pain compared to localized pain, distinct differences in circulating serum metabolites can be identified compared to control subjects [61]. Various metabolites can directly influence the perception of pain. Endocannabinoids are endogenously produced lipid-derived compounds that act as analgesics [103]. OA patients have higher levels of select endocannabinoids in the synovial fluid compared to normal individuals [104]. Thus, endocannabinoid production could be beneficial for individuals with OA to reduce pain symptoms. However, there may also be detrimental effects of increasing endocannabinoid production. Endocannabinoids use PCs as donors for their biosynthesis, resulting in the production of lysoPCs [105]. LysoPCs could promote joint pathology and ultimately pain due to metabolism to lysophosphatidic acid (LPA), an inflammatory and pain-producing signal (described in the PC-lysoPC-LPA pathway below). This could be an interesting therapeutic target for both OA symptoms and joint pathologies. Drugs used to treat pain also have effects on the metabolome. Urine analysis of rats treated with non-steroidal anti-inflammatory drugs (NSAIDs) showed altered components of the metabolome, as determined using ^1^H-NMR [62]. As NSAIDs are readily prescribed for the treatment of pain in OA, it will be necessary to evaluate how specific metabolic pathways may be affected systemically to determine potential positive and negative outcomes to NSAID use for OA.

### 3.2. Muscle Strength

Weak muscle strength is a risk factor for development and progression of knee OA [106,107]. In a mouse model of muscle wasting, gastrocnemius muscle showed changes in linoleic acid, lactate, serine, alanine, and long-chain acyl-carnitines, as measured by gas chromatography-mass spectrometry, compared to controls [66]. Several muscular dystrophy disorders also carry a variety of metabolite differences in muscle tissue, as measured using ^1^H-NMR, including BCAAs, glutamine/glutamate, histidine, acetate, propionate, fumarate, and tyrosine [63]. With regard to systemic metabolomics, a study on serum from patients with myasthenia gravis, an autoimmune neuromuscular disease resulting in muscle weakness, showed alterations of metabolite levels of a variety of glycerophospholipids with prednisone treatment [69]. In addition, there is an abundance of studies investigating circulating metabolite changes and their contributions to amyotrophic lateral sclerosis (ALS), which results in muscle weakness and paralysis in response to motor neuron degeneration. For instance, cystine and glutamic acid levels are elevated in the serum of ALS patients compared to normal subjects [64], whereas 35 metabolites in plasma could differentiate between ALS patients and healthy controls [68]. Furthermore, metabolomics analysis of blood, muscle, and brain of mice and plasma of humans suggests that arginine, proline, lysine, tryptophan, glutamate and BCAA metabolism are altered in ALS patients across tissues/fluids and species studied [65]. Additional ALS metabolomics studies are well-reviewed elsewhere [108,109]. However, muscle strength has not been well characterized with regard to its contribution to circulating plasma metabolite levels in human subjects without confounding comorbidities. In a study of a small cohort of individuals, the force exerted per muscle area by the quadriceps had a direct relationship with the systemic metabolome. Differences in PCs, lysoPCs, and various amino acids, including increased levels of leucine and isoleucine were found in weak muscle vs. control individuals [67], suggesting a possible contribution of phospholipids and BCAAs to the metabolome of OA patients with a weak muscle phenotype. Collectively, these studies suggest that there could be a link between weak muscle strength and OA incidence and progression; however, further research is needed to define metabolites from a weak muscle phenotype directly contributing to OA.

### 3.3. Obesity

There are numerous studies indicating differences in metabolite levels that are associated with BMI. For instance, in a study of normal, overweight, and obese women, BCAA concentrations in addition to kynurenine/tryptophan ratio were significantly increased in obese compared to lean and overweight individuals [83]. In a separate study, obese individuals had 10 increased and 4 decreased amino acids in circulating serum compared to healthy controls, which included BCAAs [76]. Visceral fat likely plays a role in detected metabolites correlating with BMI, as evidenced from studies of Korean individuals and women with varying levels of visceral fat [74,88]. Individuals with central obesity can also be distinguished using metabolite signatures from peripheral obesity or normal weight individuals based on serum increases in valine, isoleucine, leucine, alpha-aminoadipic acid, and C3 acylcarnitine [79]. Waist circumference in populations of dizygotic and monozygotic twins also correlates with a unique signature of serum metabolites, including valine, leucine and isoleucine [77]. Not surprisingly, BMI-based and waist circumference-based metabolite networks derived from serum metabolomic measurements are strongly correlated [86], suggesting that waist circumference and BMI could be interchangeable measures when it comes to circulating metabolites. Metabolomic analysis of plasma in Mexican American women indicates that there are seven metabolites significantly associated with BMI and six with further weight gain at a five-year follow-up [82]. In a study using three separate cohorts in the U.S. and China, a signature of 37 metabolites made up primarily of amino acids and lipids, including a variety of BCAAs, were found to be altered systemically with BMI [87]. In another study, three lysoPC analogues were found to be inversely correlated with BMI [81]. In larger cohort studies, alterations in multiple metabolites from plasma and serum were found to be associated with BMI, including BCAAs, such as valine, leucine, and isoleucine, and various lipids including lysoPC, PC, and sphingomyelins moieties [72,75,80]. Severely obese individuals also show a metabolic signature in serum, which contains a number of aromatic and branched-chain amino acids that are upregulated compared to non-obese controls [85]. Obese, metabolically-unwell individuals who have characteristics of hypertension, hyperglycemia, and dyslipidemia, showed more metabolite changes than obese, metabolically-well individuals, as compared to lean controls. In addition, obese metabolically-disparate individuals can be differentiated based on select serum metabolite levels [71,84]. Metabolic wellness, independent of BMI, can be measured by levels of BCAAs and groups of other metabolites including select short and medium chain acylcarnitines [89]. Consistently, a signature of metabolites can also differentiate obesity from metabolic syndrome [57]. Although select abnormal metabolome signatures can be attributed to obese individuals, there are cases where these signatures can also be found in normal weight subjects while some normal weight subjects can have an obese metabolome signature. This suggests a degree of heterogeneity of metabolomes of both healthy weight and obese individuals [70]. Even monozygotic twins have different plasma metabolomes likely due to environment, lifestyle, and diet, which are independent of genetics and linked to metabolic risk phenotypes [73]. The effect of environment is further supported by a study of black women from the U.S., South Africa, and Ghana, which indicates a common signature of amino acids, consisting in part of BCAAs, is present between populations while site specific obesity-related metabolites are also detected [78]. Overall, these studies suggest that increasing BMI changes the metabolome to favor metabolite patterns commonly associated with OA, possibly contributing to OA symptoms and pathogenesis. However, BMI alone may not be a minimal criterion, and metabolite levels may independently influence OA disease progression. Of particular interest, increased BCAA levels associated with many obesity-related metabolic profiles may play a vital role both systemically and locally during OA pathogenesis, as discussed below.

### 3.4. Depression

Depression contributes to the differential severity of symptoms reported by patients with OA, including pain and physical function [110]. Metabolomic analysis of individuals with depression show alterations in the serum or plasma levels of select sugars, amino acid, lipids, and purine metabolites, among others, compared to control individuals [91,93,94,95,97,99,100,101]. Sex is also a major contributing factor to changes found in the metabolome of depressive individuals. In urine, there are large differences in the metabolome between men and women with depression, with men having a larger number of metabolites showing significant changes [98]. Consequently, therapies used to treat depression can also have an impact on metabolites and change the landscape of the metabolome. For instance, selective serotonin reuptake inhibitors (SSRIs), as well as ketamine, can treat severe depression and change a variety of circulating amino acids in blood plasma, including increasing arginine in drug responders [92,96,99]. Interestingly, like in OA, arginine is reduced in individuals with depression [90]. Thus, depression may exacerbate OA symptoms or pathologies, in part, through changes in arginine metabolism, as described below. In addition, drugs known to treat depression may be valuable in OA therapy, consistent with pre-clinical animal studies showing that local injection of the SSRI fluoxetine attenuates OA progression [111]. Whether this phenomenon could be partially due to local changes to the OA metabolome requires further study. 

## 4. Metabolites and Pathways Likely Contributing to Osteoarthritis

**Figure 1:**
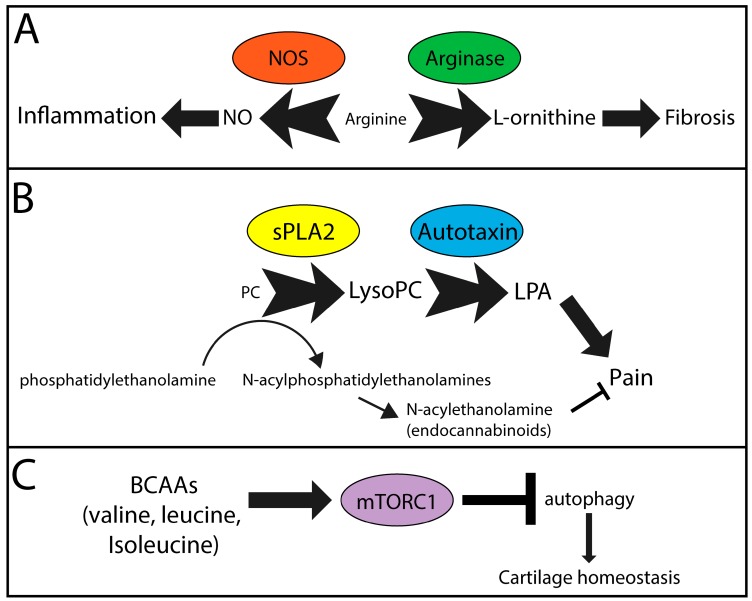
Metabolic pathways likely contributing to symptoms and pathology in OA. (**A**) Nitric oxide synthase (NOS) and arginase compete for arginine, which is reduced in the OA metabolome, to generate nitric oxide (NO) and l-orninithe, contributors to inflammation and fibrosis, respectively. (**B**) Secreted phospholipase A2 (sPLA2) catalyzes the conversion of phosphatidylcholine (PC) analogues to lysoPC analogues. Subsequent metabolism of lysoPCs via autotaxin generates lysophosphatidic acid (LPA), a signaling molecule known to promote pain. Furthermore, generation of endocannabinoids from phosphatidylethanolamines requires PC analogues, also contributing to lysoPC production. Resulting endocannabinoids function to reduce pain. (**C**) Branched chain amino acids (BCAAs) induce mammalian target of rapamycin complex 1 (mTORC1), which inhibits intracellular autophagy, a mechanism that protects cartilage homeostasis. Overall this leads to cartilage destruction and increased chondrocyte cell death. (**A**–**C**) Text size indicates concentration/activity of individual factors. Arrow/block arrow thickness indicates the likely relative contribution of each pathway in OA symptom and pathology development.

Based on clinical, anthropometric, and demographic parameters, it is clear that common metabolic pathways are altered in OA and likely contribute to local and systemic disease pathologies. These include (but are likely not limited to) arginine, lysoPC, and BCAA metabolism. A diagrammatic overview of the molecules, major enzymes, and outcomes are found in Figure 1.

### 4.1. PC-lysoPC-LPA

PCs are converted into lysoPCs by phospholipase A1 or A2 (PLA1/2) [112]. In OA, there is an increase in the expression of secreted PLA2 (sPLA2) in cells of the synovium [113], whereas human chondrocytes constitutively express sPLA2 [114]. Subsequently, autotaxin is the main enzyme that converts lysoPCs to LPA [115,116], a major contributor to pain and inflammation [117,118,119,120,121,122,123]. Increased circulating and synovial fluid levels of autotaxin have been detected in patients with OA compared to normal controls [124]. In rats, injection of LPA into the knee joint results in nerve demyelination and increased pain [125]. In a rat model of OA pain, an autotaxin inhibitor was able to attenuate pain sensitization, suggesting the activity of autotaxin and lysoPC production likely contribute to symptoms of OA [126]. Leptin is a hormone that is increased in the serum and synovial fluid of OA patients and positively correlates with BMI, increased cartilage degeneration, and female sex [127,128,129,130]. We showed that leptin increases the release of lysoPC from chondrocytes, coinciding with increased expression of autotaxin [40]. Furthermore, our in vitro data using human OA chondrocytes showed that an autotaxin inhibitor attenuated the leptin-induced chondrocyte expression of matrix metalloproteinase 13, the major cartilage extracellular matrix catabolic enzyme. Thus, autotaxin may be an OA disease and symptom-modifying target due, in part, to its ability to modify the local and systemic metabolome; however, further investigation in appropriate in vivo models is necessary to verify autotoxin inhibitors as potential multimodal therapeutics for OA. Similarly, clinical phenotypes such as obesity, muscle strength, and pain may all be influenced by changes to lysoPCs, contributing to OA pathogenesis, as described above.

### 4.2. BCAA-mTOR

Increases in the levels of valine, leucine, and isoleucine in the circulating plasma suggest that there may be dysregulated metabolism in OA patients. BCAAs are normally converted into acetyl-CoA and succinlyl-CoA, vital metabolites for energy production in the tricarboxycylic acid cycle. Increases in BCAAs are known to influence autophagy [131], specifically through the activation of mammalian target of rapamycin complex 1 (mTORC1) [132]. For instance, BCAAs increase migration of mononuclear cells via activation of mTORC1 [133], suggesting the potential for increased inflammation, an underlying pathology of OA [134]. In mouse models of OA, activation of mTOR promotes cartilage degeneration, and treatment with rapamycin attenuates cartilage degeneration when administered intra-articularly [135,136]. Altered levels of BCAAs may also contribute to insulin sensitivity and the risk of developing type II diabetes [137], as observed in OA patients. Thus, evidence suggests that an imbalance of BCAAs could lead to reduced autophagy, thereby changing cell survival and overall tissue homeostasis both systemically and in the joint, contributing to overall OA pathophysiology. Increases in BCAAs could also be contributing to or result from other clinical variables associated with phenotypes of OA, including weak muscle strength and obesity, as described above.

### 4.3. Arginine-NO/l-ornithine

There are competing metabolic pathways which utilize arginine as a substrate. Nitric oxide synthase (NOS) catalyzes the production of citrulline and nitric oxide (NO) from l-arginine [138]. Arginase produces urea and l-ornithine, which, through further metabolism, contributes to collagen synthesis and cell proliferation, factors involved in fibrosis [139,140], a pathology associated with OA. Interestingly, ornithine is increased and the ratio of arginine to ornithine is decreased in patients with OA compared to controls [22]. M2-like macrophages, typically thought of as anti-inflammatory, express arginase and promote aortic fibrosis in a mouse model of hypertension [141] and skeletal muscle fibrosis in aged mice [142]. This is in contrast to other studies that suggest that arginase-expressing macrophages are necessary to prevent Th-2-mediated fibrosis and inflammation in the liver of a schistosoma-induced fibrosis model [143]. This anti-inflammatory and anti-fibrotic role may be Th2-selective [144]. As OA is primarily Th1-mediated [145,146,147], inflammation and NO production would likely contribute to disease progression [148]. However, inducible NOS (iNOS) knockout mice have accelerated surgically-induced OA, suggesting that iNOS activity is protective against pathogenesis of surgically-induced OA [149]. Overall, based on the link of OA to cardiovascular disease, there may be a systemic push towards fibrosis while the local joint environment may have both pro-inflammatory, NO-mediated destruction, and fibrosis via arginine catabolism. As arginine is also decreased in depression, this would suggest that depression may be a consequence of changes in the OA metabolome or could exacerbate OA pathologies including fibrosis, inflammation, and pain.

## 5. Conclusions

Based on changes in the circulating metabolome, it is apparent that OA can result from and contribute to the overall systemic health of an individual. Multiple variables can contribute to changes in the metabolome in OA, including age, sex, BMI, and various co-morbidities including diabetes, depression, and cardiovascular disease. Thus, OA can be considered a complex, system-wide disease with heterogeneous etiologies where multiple variables, including the metabolome, should be weighed when caring for patients and treating the disease. Furthermore, therapies used to treat comorbidities such as depression, diabetes, and cardiovascular disease, should also be evaluated for impact in the local joint environment to determine whether local, in addition to systemic therapy, could alleviate OA pathologies and symptoms. In particular, the effects of these therapies on the local and systemic metabolome should be studied to define how these drugs influence the metabolites that cells and tissues respond to, and what types of symptom-modifying or disease-modifying responses could be expected. Further studies should concentrate on metabolic pathways known to be altered in OA including PC-lysoPC-LPA, BCAA-mTOR, and arginine-NO/l-ornithine pathways and their links to other comorbidities with common metabolite profile changes including diabetes, depression, and cardiovascular disease.

## Figures and Tables

**Table 1:** Summary of curated publications found in PubMed using the search string “metabolite AND biomarker AND osteoarthritis NOT review.”

Author	Year	Fluid/Tissue for Metabolite Detection	Species	Study Groups	Metabolite Detection Method	Reference
Anderson et al.	2018	synovial fluid	equine	septic vs. non-septic joint pathologies	^1^H-NMR	[14]
Carlson et al.	2018	synovial fluid	human	OA vs. RA vs. healthy	LC-MS	[15]
Hinata et al.	2018	synovial fluid	rat	control vs. MIA-induced OA, sham vs. meniscectomy-induced OA	LC-MS/MS	[16]
			human	OA only		
Zhang et al.	2016	plasma	human	primary OA at TKR vs. healthy control	LC-MS/MS	[17]
Jin et al.	2016	synovial fluid	human	degenerative vs. traumatic vs. infectious vs. inflammatory OA	In vivo ^1^H-MRS	[18]
Loeser et al.	2016	urine	human	OA progression vs. stable	^1^H-NMR	[19]
Mickiewicz et al.	2016	serum	mouse	sham vs. DMM; wild type vs. Integrin 1α-null; erlotinib vs. vehicle	^1^H-NMR	[20]
Hu et al.	2016	plasma	human	primary OA at TKR vs. healthy control	LC-MS/MS	[21]
Zhang et al.	2016	plasma	human	primary OA at TKR vs. healthy control	LC-MS/MS	[22]
Tufts et al.	2015	knee articular cartilage	human	primary OA at TKR	HRMAS-NMR	[23]
Zhang et al.	2015	plasma, synovial fluid	human	primary OA at TKR	LC-MS/MS	[24]
Zhai et al.	2010	serum	human	OA vs. healthy control	LC-MS/MS	[25]
Davies et al.	2009	synovial fluid, serum, cartilage	human	active OA, inactive OA, post-mortem controls	HPLC	[26]
Lamers et al.	2005	urine	human	radiographic OA vs. non-OA controls	^1^H-NMR	[27]
Basu et al.	2001	serum, synovial fluid	human	control (serum only) vs. OA vs. RA vs. ReA vs. PsA	radioimmunoassay	[28]

^1^H-MRS; proton magnetic resonance spectroscopy, ^1^H-NMR, proton nuclear magnetic imaging; DMM, destabilization of the medial meniscus; HPLC, high performance liquid chromatography; HRMAS, high-resolution magnetic angle spinning; LC, liquid chromatography; MIA; mono-iodoacetate; MS, mass spectrometry; OA, osteoarthritis; PsA, psoriatic arthritis; RA, rheumatoid arthritis; ReA, reactive arthritis; TKR, total knee replacement.

**Table 2:** Selected publications indicating metabolite changes in phenotypes related to osteoarthritis.

Phenotype	Author	Year	Fluid/Tissue for Metabolite Detection	Species	Study Groups	Metabolite Detection Method	Reference
Pain	Finco et al.	2016	urine	human	nociceptive pain vs. neuropathic pain vs. pain free	^1^H-NMR	[60]
	Hadrevi et al.	2015	serum	human	women with chronic neck pain, chronic widespread pain vs. healthy control	GS-MS	[61]
	Um et al.	2009	urine	rat	celecoxib vs. indomethacin vs. ibuprofen vs. vehicle; gastric damaged vs. undamaged	^1^H-NMR	[62]
Muscle Strength	Srivastava et al.	2018	skeletal muscle	human	Duchenne muscular dystrophy vs. Becker muscular dystrophy vs. facioscapulohumeral dystrophy vs. limb girdle muscular dystrophy vs. healthy control	^1^H-NMR	[63]
	Cieslarova et al.	2017	plasma	human	ALS vs. healthy control	CE-MS/MS	[64]
	Patin et al.	2017	Muscle and brain (mouse only), plasma	human and mouse	mSOD1*G39A-transgenic mice vs. WT mice; ALS vs. healthy control	^1^H-NMR	[65]
	Files et al.	2016	skeletal muscle	mouse	adult vs. old; sham vs. acute lung injury-induced muscle wasting	GS-MS	[66]
	Moaddel et al.	2016	plasma	human	low vs. high muscle quality in older men and women	LC-MS/MS	[67]
	Wuolikainen et al.	2016	CSF and Plasma	human	ALS and Parkinson’s disease vs. healthy control	GC-MS; LC-MS	[68]
	Sengupta et al.	2014	serum	human	myasthenia gravis prednisone treated vs. baseline	UPLC-ESI-QTOF-MS	[69]
Obesity	Cirulli et al.	2018	serum, plasma	human	metabolically obese vs. metabolically overweight vs. metabolically healthy	LC-MS/MS	[70]
	Libert et al.	2018	plasma	human	lean metabolically well vs. obese metabolically well vs. obese metabolically unwell vs. obese metabolically unwell with type II diabetes	LC-MS/MS	[71]
	Moore et al.	2018	serum	human	correlation of BMI and breast cancer risk to circulating metabolites in postmenopausal women	LC-MS/MS	[72]
	Munlandy et al.	2018	plasma	human	correlation of metabolites to cardiometabolic risk factors (including BMI, % body fat, visceral fat, subcutaneous fat) in monozygotic twins	LC-MS/MS	[73]
	Baek et al.	2017	plasma	human	low vs. high visceral fat area in a Korean cohort	LC-MS	[74]
	Carayol et al.	2017	serum, plasma	human	correlation of BMI to circulating metabolites	LC-MS/MS	[75]
	Okekunle et al.	2017	serum	human	obese vs. type II diabetes vs. metabolic syndrome vs. healthy control	UPLC-TQ/MS	[76]
	Zhong et al.	2017	plasma	human	obese vs. metabolic syndrome	LC-MS/MS	[57]
	Bogl et al.	2016	serum	human	correlation of phenotypic and obesity-related measures to metabolite levels in dizygotic and monozygotic twins	^1^H-NMR	[77]
	Dugas et al.	2016	serum	human	normal vs. obese; black women from U.S. vs. South Africa vs. Ghana	GC-TOF/MS	[78]
	Gao et al.	2016	serum	human	metabolically unhealthy centrally obese vs. metabolically healthy peripherally obese	LC-MS/MS	[79]
	Ho et al.	2016	plasma	human	correlation of BMI, waist circumference, and other metabolic traits to circulating metabolites	LC-MS/MS	[80]
	Tulipani et al.	2016	serum	human	BMI-discordant non-diabetic vs. pre-diabetic monozygotic twins	LC-MS/MS; FIA-MS/MS; ESI-MS/MS	[81]
	Zhao et al.	2016	plasma	human	correlation of metabolites to BMI and weight gain in Mexican American women	LC-MS/MS	[82]
	Boulet et al.	2015	plasma	human	lean vs. overweight vs. obese women	ESI-LC-MS/MS, ESI-MS/MS	[83]
	Chen et al.	2015	serum	human	metabolic healthy obese vs. metabolic unhealthy obese	LC-MS; GC-MS	[84]
	Gralka et al.	2015	serum	human	obese vs. normal weight	^1^H-NMR	[85]
	Floegel et al.	2014	serum	human	correlation of metabolite networks to different dietary, activity and anthropometric exposures (including BMI and waist circumference)	LC-MS/MS	[86]
	Moore et al.	2014	serum, plasma	human	correlation of metabolite levels to BMI	LC-MS/MS; GC-MS/MS	[87]
	Martin et al.	2013	plasma, urine	human	correlation of metabolites to body fat distribution in obese women	LC-MS/MS	[88]
	Batch et al.	2013	plasma	human	lean vs. overweight vs. obese	LC-MS/MS; ESI-MS/MS	[89]
Depression	Ali-Sisto et al.	2018	serum	human	major depressive disorder vs. non-depressed controls, remitted vs. non-remitted patients with major depressive disorder	LC-MS	[90]
	Kawamura et al.	2018	plasma	human	major depressive disorder vs. mentally healthy controls	CE-TOF/MS	[91]
	Moaddel et al.	2018	plasma	human	major depressive disorder vs. healthy controls, ketamine vs. placebo	LC-MS/MS	[92]
	Zheng et al.	2017	plasma	human	major depressive disorder vs. healthy controls	^1^H-NMR	[93]
	Ali-Sisto et al.	2016	serum	human	major depressive disorder vs. non-depressed controls	LC-MS/MS	[94]
	Liu et al.	2016	plasma	human	healthy controls vs. major depressive disorder, melancholic depressed, anxious depressed	LC-MS/MS, GC-MS	[95]
	Rotroff et al.	2016	plasma	human	baseline vs. post-treatment of patients with major depressive disorder treated with placebo, ketamine, or esketamine	LC-MS/MS, GC-TOF/MS	[96]
	Setoyama et al.	2016	plasma	human	correlation of metabolites to depression severity in patients with psychiatric disorders, drug-free major depressive disorder, or bipolar disorders; medicated major depressive disorder and bipolar disorders	LC-MS	[97]
	Zheng et al.	2016	urine	human	major depressive disorder vs. healthy controls, women vs. men	^1^H-NMR, GC-MS	[98]
	Woo et al.	2015	plasma	human	healthy controls vs. major depressive disorder patients baseline vs. major depressive disorder patients 6-weeks post SSRI treatment	LC-MS/MS	[99]
	Zheng et al.	2012	plasma	human	drug-naïve first episode depression vs. healthy controls	^1^H-NMR	[100]
	Paige et al.	2007	plasma	human	remitted depressed vs. non-remitted depressed vs. non-depressed older adults	GC-MS	[101]

^1^H-NMR, proton nuclear magnetic imaging; ALS, amyotrophic lateral sclerosis; BMI, body mass index; CE, capillary electrophoresis; CSF, cerebrospinal fluid, ESI, electrospray ionization; FIA, flow injection analysis; GC, gas chromatography; LC, liquid chromatography; MS, mass spectrometry; QTOF, quadrupole time of flight; SSRI, selective serotonin reuptake inhibitor; TOF, time of flight; UPLC, ultra-performance liquid chromatography; WT, wild-type.
